# Can Digital Finance Promote Peak Carbon Dioxide Emissions? Evidence from China

**DOI:** 10.3390/ijerph192114276

**Published:** 2022-11-01

**Authors:** Mao Wu, Jiayi Guo, Hongzhi Tian, Yuanyuan Hong

**Affiliations:** 1 School of Economics and Management, Northwest University, Xi’an 710127, China; 2 School of Environment and Spatial Informatics, China University of Mining and Technology, Xuzhou 221116, China; 3 School of Management Science and Engineering, Nanjing University of Information Science and Technology, Nanjing 210044, China

**Keywords:** digital finance, carbon dioxide emissions, energy industrial structure, carbon reduction, green innovation

## Abstract

This paper uses Chinese provincial panel data from 2011 to 2019, measures CO_2_ emissions of provinces in China using the IPCC method, and explores the impact of digital finance on CO_2_ emissions through the SAR model and SDM. Empirical study shows that digital finance significantly reduces CO_2_ emissions. Digital finance reduces CO_2_ emissions by promoting energy industrial structure transformation and spreads to surrounding areas through spillover effects, contributes to increasing green patents granted and thus reduces regional CO_2_ emissions, advances the green technological progress and therefore inhibits CO_2_ emissions, but reduces the green technological progress in surrounding areas and increases CO_2_ emissions due to the siphon effect. With the development of digital finance itself, the higher the level of financial regulation, green development and the green finance index, the better the effect of digital finance on CO_2_ emission reduction. Additionally, digital finance significantly reduces CO_2_ emissions in the south of China.

## 1. Introduction

The increase in global extreme weather in recent years has resulted in serious damages to the economic and social development of countries around the world. The International Panel on Climate Change has pointed out that the increasing global anthropogenic greenhouse gases emissions (GHGs), especially carbon dioxide, have led to many environmental problems including global warming [1], which has been one of the major challenges for human well-being [2,3]. The International Panel on Climate Change has pointed out that the increasing global carbon emissions have led to many environmental problems including global warming [4]. As the world’s largest developing country and second largest economy, since the implementation of the “Reform and Opening-up” policy in 1978, China has made significant progress in the aspects of economic growth, industrialization and social progress. However, according to the Global Carbon Atlas (GCA), The CO_2_ emissions in China increased from 1455 million tons in 1978 to 10,175 million tons in 2019, making it the world’s highest emitter since it overtook the United States in 2006. The current stage of China’s economic developing mode that relies on the extensive input of material capital and labors determines its energy consumption structure and carbon emissions. However, as the largest carbon dioxide-emitting economy in the world, China’s process of peak carbon emissions plays an important role in limiting global temperature rise [5]. Under this circumstance, according to the intended nationally determined contributions (INDC), the Chinese government announced its commitment to attain the CO_2_ emissions peak around 2030 and reduce CO_2_ emissions per unit of the gross domestic product (GDP) by 60–65% from the level of 2005 [6]. 

Given the significant impact of carbon emissions on global climate change and human social activities, the Chinese government committed to the US–China Joint Communique on Climate Change issued in November 2014 to peak and stop growing greenhouse gas emissions from fossil energy combustion activities such as coal, oil, and natural gas; industrial production processes; as well as land use change and forestry by 2030. At the general debate of the 75th session of the UN General Assembly, President Xi Jinping also proposed that China will improve the nationally determined contributions and adopt tougher policies and measures to strive to reach peak carbon emissions by 2030 and carbon neutrality by 2060. Facing such great pressure from CO_2_ emission reduction, since the mode of Chinese carbon reduction was proposed, it immediately received a lot of attention from the international and domestic communities. 

In the meanwhile, at present, artificial intelligence, big data, and the blockchain are increasingly applied in the modern industrial system, which not only contribute more and more importantly to economic growth, but also greatly improve people’s quality of life. In the process of forming the modern economic system, financial support guided by the development of digital finance also plays a pivotal role [7]. Digital finance has significantly expanded the scope of financial coverage, lowered the threshold and cost of financial services, and increased the efficiency of financial services [8]. Over the past decade, digital finance business in China has grown by leaps and bounds, contributing to not only economic growth, but also various aspects of economic development, including greenhouse gas emissions reduction [9]. Through upgrading the industrial structure, digital finance significantly reduces industrial pollution [10] and carbon emissions [11], alleviates financial constraints and increases R&D investment, improves the efficiency of green investment [12], enhances regional agricultural green total factor productivity [13] and the level of regional openness to the outside world, benefits the coordination of regional development, and promotes high-quality development [14].

Overall, there has been plenty of research on carbon emissions and digital finance as well as their relationships. However, most of the existing literature does not distinguish between physical and chemical changes in energy consumption; it conducts CO_2_ emissions accounts through the numerical value of energy consumption, which tends to overestimate emissions. Additionally, a lot of literature has studied the relationship between digital finance and carbon emissions, but there is a lack of research that identifies the causality between digital finance and carbon emissions excluding endogeneity and examining the mechanism from a spatial perspective.

Therefore, this paper shifts the perspective to adopt more accurate indicators and qualitative parameters to measure carbon emissions. At the same time, this paper conducts causal inferences on whether digital finance effectively suppresses regional carbon emissions, which takes issues such as endogeneity and so on into consideration under the spatial perspective, and further explores the mechanisms of digital finance affecting carbon emission reduction under the spatial perspective, as well as the heterogeneity of digital finance on carbon emissions in terms of the different regions, digital finance itself, financial regulation, and green development as well as green finance development. The in-depth study of these issues will help us seek the channel of financial support for peak carbon dioxide emissions and test the role of digitalization on financial development, so as to provide recommendations for decision-making on the development of digital finance and help to peak carbon dioxide emissions in the context of the digital economy.

## 2. Theoretical Analysis

### 2.1. Theoretical Analysis of Reducing Carbon Emissions by Digital Finance

The foothold of carbon emission reduction is sustainable development, “satisfying the present without harming the future”, and improving people’s quality of life. Therefore, we should reduce energy consumption and carbon emissions through energy structure transformation and green technology innovation without reducing production capacity, so as to achieve green and sustainable development. Developing finance is a reliable path to achieving carbon emission reduction [15]. Digital finance, referring to the financial business carried out by banks, other traditional financial institutions, and Internet enterprises using digital technology [16], leads to the profound transformation of financial mode that enables businesses to access financial services through digital channels [17]. The greatest advantage of digital finance is the use of innovative technology such as information technology, big data technology, and cloud computing to reduce financial transaction costs, expand the scope of financial services, and improve financial accessibility [18], which has played a significant role in sustainable development [19] and low-carbon emission reduction [20]. 

However, high-tech enterprises with low energy consumption and the technological innovation on which they depend are characterized by high investment, long research periods, and uncertainty [21], coupled with information asymmetry [22]. Here, commercial banks affirm loans for technological innovations as riskier when evaluating. At the same time, commercial banks tend to avoid high-risk projects due to regulations that require them to be risk-averse, which makes it difficult for enterprises to obtain loans for technological innovation projects, thus inhibiting technological innovations. Digital finance, relying on a large amount of individual behavior data and other information precipitated by lenders on the Internet, can build credit evaluation models by means of big data analysis [23], and alleviate the financial constraints of enterprises [24], especially in the sectors of emerging industry with low carbon emissions, through risk assessment of big data [25]. On the other hand, the regulatory risk requirements of Internet financial platforms are not strict as those of banks. Thus, they can do better in evaluating the credit and future profitability of these technological innovation behaviors and undertake a higher risk than banks when providing lending to these enterprises. Thus, they can obtain funds for debt financing, so as to promote the green technology innovations of enterprises. Therefore, digital finance has greatly alleviated the financial constraints of enterprises, stimulated green technological innovation behaviors [26], and assisted enterprises with CO_2_ emissions reduction. As the essential micro-component of regional market participation, the reduction of enterprises’ CO_2_ emissions would help to suppress regional carbon emissions to a great extent. Accordingly, this paper puts forward the following hypothesis:

**H1.**  *Digital finance alleviates the financial constraints of enterprises, thus reducing regional CO_2_ emissions.*

### 2.2. Theoretical Analysis of Digital Finance for Energy Structural Transformation

Energy structure refers to the composition and proportion of various types of energy in the total production or consumption of energy. Digital finance provides important support for the development of low-energy-consumption industries and the transformation of the energy industrial structure. Traditional energy-intensive industries are mostly state-owned enterprises with large scale, which can obtain loans through their high mortgage assets in the financial system with banking as the main financial intermediary participants, while enterprises devoted to green technological innovations and their innovative projects are excluded by the traditional financial system, making it difficult for enterprises to develop and land the projects. By alleviating the financial constraints of green innovative enterprises and projects, digital finance helps the development of relatively small-scale green technological innovative [26] so as to improve the output ratio of regional low-energy-consumption industries, improve the efficiency of capital investment, strengthen the function of capital allocation, promote the transformation of funds to low-energy-consumption and high-efficiency industries, eliminate backward industries, optimize the reallocation of funds among industries, and promote the transformation of regional energy industrial structures, so as to achieve the goal of reducing carbon emissions. Based on the analysis above, this paper puts forward this hypothesis:

**H2.**  *Digital finance can reduce carbon emissions by promoting the transformation of the energy industrial structure.*

### 2.3. Theoretical Analysis of Digital Finance Promoting Green Technological Progress

Viewing the process of global carbon emission reduction, the reasons why some countries realize the peak carbon dioxide emissions and carbon neutrality faster is that they are more energy efficient and developed in innovative low-carbon technology. Reducing carbon emissions requires strong innovation capacity. Therefore, achieving technological innovation is one of the most important ways of achieving carbon emission reduction [27]. Accelerating the technological progress of industries and promoting green innovations are vital for reducing carbon emissions. Finance will undoubtedly provide the necessary funding for the innovative behaviors upon which technological advancement depends. Through investing in high-quality innovative industries, finance is considered one of the most important factors in promoting sustainable economic development [28]. The excellent information collection and processing capability of digital finance can provide better play to the functions of filtering and screening information as well as evaluating risks, so that it can help mitigate information asymmetry [22]. Relying on digital technologies such as cloud computing, big data, and mobile Internet, the risk control, information processing, and monitoring systems that digital financial platforms depend on could efficiently and accurately mine and collect the credit and investigation data of funding demanders. By giving full play to digital advantages, digital finance helps to build a credit evaluation model and a more comprehensive credit investigation system, breaks through the limitations of traditional finance, mitigates information asymmetry between credit providers and demanders, and improves information transparency. By widely absorbing financial resources in the market, digital finance would convert them into accurate and effective information and data, reducing the financing costs of innovative behaviors and technological progress as well as solving the financing troubles of innovative subjects. Long-term and high-risk green projects could receive much-needed financial support, which would improve the utilization efficiency of resources [11], so as to support the development of green innovative behaviors and technological progress, thus reducing carbon emissions through green innovation and technological progress. Therefore, this paper puts forward the hypothesis:

**H3.**  *Digital finance can reduce carbon emissions by promoting green innovation and technological progress.*

**Figure 1:**
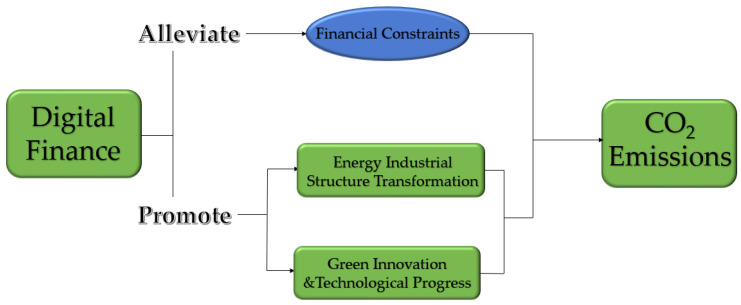
Graph of mechanisms of digital finance affecting CO_2_ emissions.

The Hypothesis of this paper can be seen in Figure 1.

## 3. Model Setting, Variable Selection, and Data Source

### 3.1. Accounting of Carbon Dioxide Emissions

#### 3.1.1. Accounting Method

According to the carbon dioxide emissions accounting methods provided in the *Guidelines for the Preparation of Provincial Greenhouse Gas Inventories* and the *Guidelines for National Greenhouse Gas Inventories* of the IPCC, the calculation method for CO_2_ emissions is shown in the following formula:

*PC_i_* = ∑*_ij_M_ij_* × *EF_i_* (1)

where *PC* stands for carbon dioxide emissions, *i* stands for the subscript of the province, *j* stands for the subscript of the energy type, *M* stands for energy consumption, and *EF* stands for the carbon emissions factor.

#### 3.1.2. Emission Factors

The calculation formula for the carbon emissions factor is as follows:

*EF_ij_* = *C_j_* × *O_ij_* × 44/12 (2)

where *C* represents the carbon content; *O* represents the carbon oxidation rate of the energy source. Due to the slightly different energy sources and accounting boundaries of power industry carbon accounting in different regions, the energy carbon oxidation rate is different in different regions, so the baseline carbon emissions factors of the power grid are different.

#### 3.1.3. Carbon Content

The calculation formula for carbon content is as follows:

*C_j_* = *NCV_j_* × *H_j_* (3)

where *NCV* represents the low calorific value of generation of fuel *j*; *H_j_* represents the carbon content per unit calorific value of fuel *j*.

**Figure 2:**
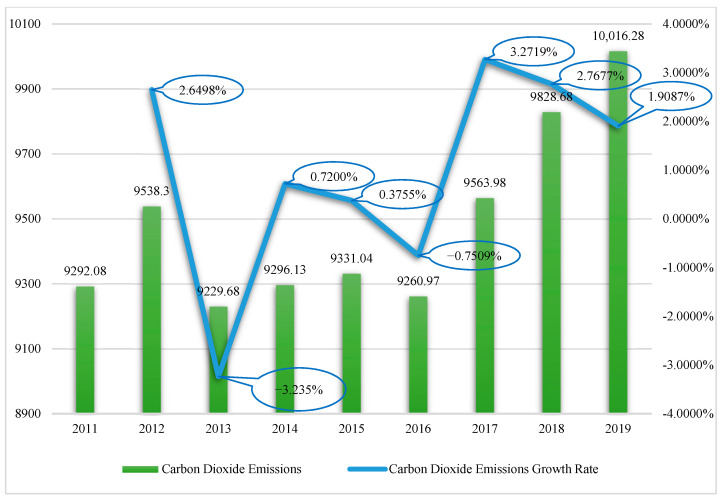
Graph of annual carbon dioxide emissions trend in China.

The carbon content per unit calorific value and carbon oxidation rate of various energy sources refer to the IPCC *Guidelines for National Greenhouse Gas Inventories*, and the default values of carbon content and oxidation rate in are expressed as calorific value units. Therefore, it is necessary to convert the physical amount consumed by various energy sources into the corresponding average low calorific value according to the default value of net heating given in the guide. The default values of average low calorific value are carbon content per unit, calorific value, and the carbon oxidation rate of various energy sources. The CO_2_ emissions in China from 2011 to 2019 are shown in Figure 2.

### 3.2. Variables Selection

#### 3.2.1. Explained Variable

Carbon dioxide emissions (*CO*). The CO_2_ emissions from each provincial region of 30 provinces, cities, and autonomous regions (except Hong Kong, Macao, Taiwan, and Tibet) were selected as the index of the explained variable. The annual CO_2_ emissions from 2011 to 2019 were calculated using IPCC inventory method. We then took the logarithm of the initial value to obtain the explained variable. 

#### 3.2.2. Core Explanatory Variable

Digital Finance (*DFI*). Adopted the *Digital Financial Inclusion Index* released by the Digital Finance Research Center of Peking University [16]. 

#### 3.2.3. Mediating Variable

For the energy industrial structure (*ES*), with reference to Ma and Zhang [29], we constructed the energy industrial structure index:

(4) $${\mathrm{ES}}_{\mathrm{it}}=\frac{\sum_{i=1}^{8}HCI_{it,j}}{GDP_{it}}$$

*ES_it_* is the energy industrial structure of region *i* in year *t*. *GDP_it_* denotes the regional gross production in region *i* in year *t*, *HCI_it,j_* denotes the output value of the energy-intensive industry *j* in year *t* of region *i.* The eight selected industries were the electricity, heat production and supply industry, petroleum processing, coking and nuclear fuel processing industry, ferrous metal smelting and rolling processing industry, non-metallic mineral products industry, coal mining and washing industry, chemical raw materials and chemical products manufacturing industry, non-ferrous metal smelting and rolling processing industry, and the paper and paper products industry.

Green innovation (*GI*), the number of green patents granted in year *t* in region *i*, was chosen to characterize the level of green innovation (unit: 10^4^).

Green technological progress (*TP*), the frontier technological progress rate in green total factor productivity was selected. Firstly, we adopted the following transcendental logarithm stochastic frontier production function [30]:

*lny_it_
*=* ln *[*x_it_
*(*t*),* t*] +* ζ_t_* **=* c_*0*_
*+* c_l_lnL_it_
*+* c_k_lnK_it_
*+* c_e_lnE_it_
*+* c_t_t *+* c_lk_lnL_it_lnK_it_
*+* c_le_lnL_it_lnE_it_
*+* c_ke_lnK_it_lnE_it_
*+* c_lke_lnL_it_lnK_it_lnE_it_
*+* c_tl_tlnL_it_
*+* c_tk_tlnK_i_
*+** *c_te_tlnE_it_
*+* c_t_^*2*^t^*2*^
*+* c_l_^*2*^ln^*2*^L_it_
*+* c_k_^*2*^ln^*2*^K_it_
*+* c_e_^*2*^ln^*2*^E_it_
*+* ζ_t_* (5)

where *i* denotes the subscript of the region; *t* denotes the subscript of the year; *y* denotes real regional GDP, obtained by deflating regional GDP through the regional GDP index; and *K* denotes capital stock, using gross fixed capital formation, obtained by deflating the fixed asset investment price index using 2005 as the base year to obtain real gross fixed capital formation from 2006 to 2019, which is accounted for under the perpetual inventory method. Using a depreciation rate of 9.6% for fixed assets [31], the actual gross fixed capital formation in 2006 was taken as the capital stock in 2006, and then the capital stock of the previous year was depreciated and added to the actual gross fixed capital formation in the current year as the capital stock in the current year. *L* represents the employed population of each region; *E* represents the energy consumption, and the energy used in each region was converted into standard coal through the conversion coefficient as the energy consumption of the region this year; and *ζ_t_* stands for random term. We estimated the coefficient in Equation (5) through OLS, and then calculated the frontier technological progress rate *TP* as

(6) $$TP=\frac{\partial ln\left[x_{it}\left(t\right),t\right]}{\partial t}=c_{t}+c_{tl}lnL_{it}+c_{tk}lnK_{i}+c_{te}lnE_{it}+2{c_{t}}^{2}t$$

#### 3.2.4. Covariables

In order to prevent the problem of omitting variables, this paper refers to the previous studies [11,20,32], and chooses the following covariables: 

The economic development degree of each province (*RGDP*), expressed by regional GDP per capita of each provincial administrative unit.

Regional population (*POP*), expressed by the logarithm of the regional resident population of each provincial administrative unit.

Fiscal expenditure on environmental protection (*FEP*), adopting the logarithm of regional fiscal expenditure on environmental protection.

The total volume of import and export (*IX*), expressed by the logarithm of regional total import and export.

Industrial structure (*IS*), constructed using the industrial structure index [33]:

(7) $${\mathrm{Ind}}_{\mathrm{it}}=\sum_{j=1}^{3}j\times Ipj_{it},\;j\in Z$$

*Ind_it_* is the industrial structure index of the region *i* in the year *t*, and *IPj_it_* is the proportion of the output of the industry *j* of the regional GDP of the *i*-th region in the year *t*. The index is used to represent the regional industrial structure: 

Urban greening rate (*UG*), which adopts the urban greening rate of each province and city is expressed.

### 3.3. Econometric Model Setting

Considering the possible spatial correlation between CO_2_ emissions and the digital financial inclusion index. This paper conducts Moran index tests for spatial correlation between the digital financial inclusion index and CO_2_ emissions. Table 1 reports the results of spatial correlation tests. Where the spatial weighted matrix includes the provincial adjacency matrix, the provincial capital distance matrix and the spatial geographic reverse matrix. It can be surmised from the table that there is a significant spatial correlation between CO_2_ emissions and the digital financial inclusion index no matter which spatial weighted matrix is chosen. However, the spatial geographic reverse matrix shows the most significant spatial correlation between CO_2_ emissions and the digital financial inclusion index. Therefore, we adopted the spatial econometrics model and selected the spatial geographic reverse matrix in the baseline regression. The provincial adjacency matrix and the provincial capital distance matrix were adopted in the robustness test.

Furthermore, in order to make sure what model ought to be set, this paper adopted LR tests, and the results show that the probability of the SAR (spatial autoregression model) nested in an SDM (spatial Durbin model) is about 14%, while the probability of the SEM (spatial errors model) nested in an SDM is less than 1%. Thus, we rejected the hypothesis of SEM nested in SDM and there has no obvious evidence of whether SAR is nested in SDM or not. Therefore, the baseline regression model was set as follows:

*CO_it_* = *α*_01_*DFI_it_* + ***X_it_γ*****_0_** + *α*_02_***W****CO_it_* + *u_t_* + *λ_i_* + *ε_it_* (8)

*CO_it_* = *α*_11_*DFI_it_* + ***X_it_γ*****_1_** + *α*_12_***W****CO_it_* + *α*_13_***W****DFI_it_* + ***WX_it_γ*****_1_** + *u_t_* + *λ_i_* + *ε_it_* (9)

where *i* is the regional subscript of province and *t* is the time subscript of year. *CO* is the indicator of CO_2_ emissions, *DFI* is the digital financial inclusion index, ***X_it_*** is the covariables matrix, *u_t_* is the time fixed effect, *λ_i_* is the province fixed effect, and *ε_it_* is the random error term. Formula (8) is the SAR model and Formula (9) is the SDM, which are both adopted in the baseline regression.

Meanwhile, in order to better explore how digital finance promotes the reduction of CO_2_ emissions, this paper explored the mediating mechanisms. Owing to the controversies of the mediating effect model [34], this paper directly tested the causal relationship between the mechanism variables and the explanatory variables. The model is set as follows:

*Med_it_* = *β*_01_*DFI_it_* + ***X_it_η*****_0_** + *β*_02_***W****Med_it_* + *u_t_* + *λ_i_* + *ε_it_* (10)

*Med_it_* = *β*_11_*DFI_it_* + ***X_it_η*****_1_** + *β*_12_***W****Med_it_* + *β*_13_***W****DFI_it_* + ***WX_it_η*_2_** + *u_t_* + *λ_i_* + *ε_it_* (11)

*CO_it_* = *υ*_01_*Med_it_* + ***X_it_ϖ*_0_** + *υ*_02_***W****CO_it_* + *u_t_* + *λ_i_* + *ε_it_* (12)

*CO_it_* = *υ*_11_*Med_it_* + ***X_it_ϖ*****_1_** + *υ*_12_***W****CO_it_* + *υ*_13_***W****Med_it_* + ***WX_it_ϖ*_3_** + *u_t_* + *λ_i_* + *ε_it_* (13)

where *Med_it_* denotes the mediating variable. If *υ*_01_ is significantly not 0, it means that the mediating variable is significantly correlated with the explained variable, while when *β*_01_ is significantly not 0, namely, digital finance has a significant impact on the mediating variable, so it can be taken for granted that when using the SAR model, the mediating mechanism that digital finance affects CO_2_ emissions through the mediating variable holds. Additionally, if *β*_11_ and *υ*_11_ are significantly not 0, it indicates that the mediating mechanism holds when using the SDM, while if *υ*_01_ and *υ*_13_ are significantly not 0, it means that digital finance influences CO_2_ emissions in the surrounding areas by affecting the mediating variable in surrounding areas.

### 3.4. Data Sources

The data from the digital financial inclusion index used in this paper comes from *The Peking University Digital Financial Inclusion Index of China* issued by the research center of digital finance of Peking University. The original data for calculating carbon dioxide emissions are from the energy balance tables (physical quantities) of 30 provinces, cities, and autonomous regions in the *China Energy Statistical Yearbook* issued from 2012 to 2020. The data on the output value of energy-intensive industries, regional gross output value, total fixed capital formation and employed population of energy-intensive industries are from the official website of the National Bureau of Statistics, the data on green patents are from the official website of the China Patent Database, and the data on covariables are from the official website of the National Bureau of Statistics. The sample of CO_2_ emissions studied in this paper comprised 30 provinces, municipalities, and autonomous regions except Hong Kong, Macao, Taiwan, and Tibet from 2011 to 2019, and the explanatory variables comprised 30 provinces and municipalities directly under the central government except Hong Kong, Macao, Taiwan, and Tibet from 2011 to 2019. Table 2 shows the descriptive statistics of the main variables.

## 4. Empirical Analysis Results

### 4.1. Baseline Regression

Table 3 reports the regression results of the effect of the digital financial inclusion index on CO_2_ emissions. It can be seen from the table that without adding covariables, the inhibitory effect of the digital financial inclusion index on CO_2_ emissions was significant at the 1% level when using the SAR model and at 5% when using the SDM, indicating that digital finance significantly inhibits CO_2_ emissions. After adding covariables, the regression coefficient was significantly negative at the 1% level when adopting the SAR model and at 5% when using the SDM as well. As for the spatial term of the digital financial inclusion index, it showed no significance to the coefficient. As for covariables, regional population is significantly correlated with CO_2_ emissions at 10%. The spatial lag term of explained variable is negatively correlated with itself. 

### 4.2. Endogenous Tests

The baseline regression above verifies the inhibitory effect of the digital financial inclusion index on CO_2_ emissions, but there may be endogenous problems in the process of model causality identification. Although the model has set covariables, there may still be other unobservable factors that aren’t included in the model that affect the explained variable that have nothing to do with digital finance. Therefore, in order to better identify the relationship between the digital financial inclusion index and CO_2_ emissions, this paper conducted endogenous analysis using the 2SLS approach, using the spherical distance between the city and Hangzhou [35] and dividing by the sum of the total digital financial inclusion index of the year for the whole country (except the province itself) as the instrumental variable (*DIS*). Additionally, we referred to Liu et al. [36], using the total amount of postal and telecommunications services in each region in 1996, the year before Chongqing was established as the municipality, multiplied by the density of Internet broadband access ports in the previous year as the instrumental variable (*TS*). Panel A of Table 4 reports the first stage results of the 2SLS regressions, where the regression of two instrumental variables from the digital financial inclusion index in the first stage regression were significant at the 1% level when using the SAR model. While using the SDM, the instrumental variable *DIS* was significantly positively correlated with the digital financial inclusion index at 10% and the instrumental variable TS was significantly positively correlated with the digital financial inclusion index at the 1% level.

Panel B of Table 4 reports the results of the 2SLS second stage regression, from which it can be seen that the digital financial inclusion index estimated by *TS* showed a significant negative correlation with CO_2_ emissions at 5% when using the SAR model and SDM, so did the digital financial inclusion index estimated by *DIS* when adopting SDM, and it showed a significant inhibitory effect on CO_2_ emissions at 1% when using the SAR model. The K-P LM statistic is 17.212, which corresponds to the *p*-value of 0.0002, so the instrumental variables are identifiable. The Cragg–Donald Wald F statistic of *TS* is 20.135, K-P Wald F statistic of it is 18.117, greater than 10% maximal IV size Stock–Yogo weak ID test critical value of 16.38. Thus, *TS* was not weakly correlated with the digital financial inclusion index. The Cragg–Donald Wald F statistic of *DIS* is 12.273, greater than 15% maximal IV size Stock–Yogo value of 8.96, and K-P Wald F statistic of it is 6.488, greater than 25% maximal IV size Stock–Yogo value. The Hansen J statistic is 17.862, corresponding to the *p*-value of 0.0000, thus the instrumental variables were not overidentified, which means they are exogenous.

Additionally, considering the possible existence of reverse causality biasing the model estimation, this paper also performed the dynamic spatial panel model test. Panel C of Table 4 shows the results. Columns (1), (2), and (3) show the regression results that lag all explanatory variables by one period, i.e., the sample of CO_2_ emissions is from 2012 to 2020, and the data is still from the *China Energy Statistical Yearbook*. Columns (4), (5), and (6) are the results of regressions that lag all explanatory variables by one period and then takes CO_2_ emissions, which is lagged one period as the explanatory variable. The results of the regressions on Panel C of Table 4 indicate that the digital financial inclusion index reduces carbon emissions at no less than a 5% significance level.

At the same time, to better exclude endogeneity and test whether digital finance reduces CO_2_ emissions, this paper referred to Song et al. [37], selected “*Promoting the Development Plan for Financial Inclusion (2016–2020)*” as the exogenous policy shock and conducted the spatial difference-in-difference (DID) estimation. We set the *TREAT* variable, took it for 1 when the numerical value of the digital financial inclusion index in the province was among the top 15 provinces in China in 2015, and otherwise took it for 0. 

**Figure 3:**
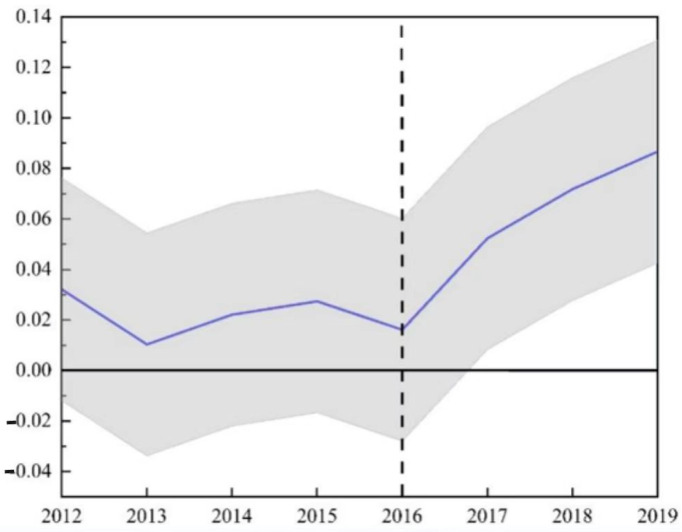
Parallel trend test.

Also, we set the *POST* variable and took it for 1 when the year was greater than or equal to 2016, otherwise taking it for 0. Finally, we let *DID* = *TREAT* × *POST*. Before starting the estimation, we endeavored to perform the parallel trend test, and Figure 3 reports the result of the parallel trend test. The vertical axis represents CO_2_ emissions, and the horizontal axis represents the year. From the figure, we can see that the regression coefficient of CO_2_ emissions and the year before the policy implementation is not significantly not 0, while this regression coefficient is significantly greater than 0 at 5% after the policy implementation, so there is a significant difference in carbon emissions before and after the policy implementation, the parallel trend test passed.

Thus, this paper set the spatial DID model as follows:

*CO_it_* = *λ*_01_*DID_it_* + ***X_it_Γ*****_0_** + *λ*_02_***W****CO_it_* + *u_t_* + *λ_i_* + *ε_it_* (14)

*CO_it_* = *λ*_11_*DFI_it_* + ***X_it_Γ*****_1_** + *λ*_12_***W****CO_it_* + *λ*_13_***W****DFI_it_* + ***WX_it_Γ*****_2_** + *u_t_* + *λ_i_* + *ε_it_* (15)

The results of the spatial DID model estimation are reported in Table 5, the result of the SAR–DID model estimation is listed in column (1) and the SDM–DID model estimation is listed in (2) and (3), from which it can be seen that the *DID* variable still shows a significant reduction in CO_2_ emissions even though CO_2_ emissions rise significantly after the policy is implemented, so the digital finance represented by this exogenous shock has a significant reduction effect on CO_2_ emissions. Further, considering that the data of CO_2_ emissions between different regions may not satisfy the parallel trend before and after policy implementation, this paper referred to Qi et al. [38] to test for policy shock by using the spatial DDD model, setting the variable *SECT* and taking it for 1 when the province was in the top 15 of China in terms of CO_2_ emissions in 2015, otherwise taking it for 0, and the variable *DDD* = *DID* × *SECT*. After that, the spatial *DDD* model was set as follows:

*CO_it_* = *θ*_00_ + *θ*_01_*DDD_it_* + *θ*_02_*DID_it_* + *θ*_03_*TREAT_it_ × SECT_it_* + *θ*_04_*POST_it_ × SECT_it_* + *θ*_05_*TREAT_it_* + *θ*_06_*POST_it_* + *θ*_07_*SECT_it_* + ***X_it_Φ*_0_** + *θ*_08_***W****CO_it_* + *ε_it_* (16)

*CO_it_* = *θ*_10_ + *θ*_11_*DDD_it_* + *θ*_12_*DID_it_* + *θ*_13_*TREAT_it_ × SECT_it_* + *θ*_14_*POST_it_ × SECT_it_* + *θ*_15_*TREAT_it_* + *θ*_16_*POST_it_* + *θ*_17_*SECT_it_* + ***X_it_Φ*_1_** + *θ*_18_***W****CO_it_* + *θ*_19_***W****DFI_it_* + ***WX_it_Γ*****_2_** + *ε_it_* (17)

Given the possible multicollinearity problem of fixed effects with *POST*, *SECT*, and their interaction term, we used random effect in the spatial DDD model. Table 5 also reports the regression results of the spatial DDD model, showing that the *DDD* variable is negatively correlated with CO_2_ emissions at 5% significance level when using the SAR-DDD model, while the *DDD* variable is negatively correlated with CO_2_ emissions at the 1% significance level when adopting the SDM–DDD, so the exogenous shock test passed. Thus, the endogenous test passed, the effect of the digital financial inclusion index on CO_2_ emissions reduction still held robustly after considering possible omitted variables such as reverse causality, which would cause estimation bias.

### 4.3. Robustness Tests

In order to verify the robustness of this conclusion, the following tests were also carried out:

Replaced estimation model test: The models selected for the test included the Pooled OLS, panel data fixed effect model, differential GMM (DIF–DID) model, SYS-GMM model, spatial error model (SEM) and spatial autocorrelation (SAC) model. The regression results of the replaced model tests are shown on Panel A of Table 6, indicating that the digital financial inclusion index suppresses CO_2_ emissions at the 5% significance level when using the Pooled OLS, differential GMM model and SYS-GMM model, while the digital financial inclusion index suppresses CO_2_ emissions at 1% when adopting the panel data fixed effect model, SEM and SAC model. Thus, after taking the possible model setting bias which would affect conclusions into account, the digital financial inclusion index still significantly suppresses CO_2_ emissions.

Replaced spatial weighted matrix test: We adopted the provincial adjacency matrix and the provincial capital distance matrix to replace the spatial geographic reverse matrix in the baseline regression. Panel B of Table 6 shows regression results of the replaced spatial weighted matrix robustness test, showing that except for the adoption of SDM with the provincial capital distance matrix, the digital financial inclusion index reduces CO_2_ emissions at the 5% significance level, the regression results from the rest of the models are negatively correlated at the 1% significance level between two variables. Thus, the replacement of the spatial weight matrix robustness test was performed.

Replaced explained variables: Using the raw value of CO_2_ emissions (unit: 10^7^ t) to replace the logarithmic value in the baseline regression, the regression results shown in Panel C of Table 6 indicate that the digital financial inclusion index is negatively correlated at the 5% significance level with the raw value of CO_2_ emissions. In the meanwhile, this paper used data from the CEADs China Sub-Provincial CO_2_ emissions Database to represent the indicator of CO_2_ emissions, and the regression results that are also shown in Panel C of Table 6 denote that it is negatively correlated with the core explanatory variable at 1%, so the robustness test of the replacing variable passes. Thus, after considering possible endogeneity issues, the spatial weighted matrix and variable forms, as well as data sources that would bias the estimation results, the effect of digital finance on carbon emission reduction still holds robustly, thus verifying H1 of this paper.

### 4.4. Mediating Mechanisms Tests

The previous sections of this paper verified the significant effect of digital finance on carbon emission reduction through causal inference from the spatial perspective. Next, this paper goes on to test the mediating mechanism behind digital finance contributing to carbon emission reduction from a spatial perspective

Panel A of Table 7 reports the reduction effect of digital finance on CO_2_ emissions through energy structure transformation. As can be seen from the table, digital finance significantly reduces the proportion of energy-intensive industries’ output to regional GDP at 1% when using the SAR model, while the effect is significant at 10% in the local area when adopting the SDM, and significant at 1% in the surrounding areas, indicating that the transformation of the energy industrial structure driven by digital finance has a spillover effect to the surrounding areas. Thus, digital finance reduces CO_2_ emissions in the local and surrounding areas by promoting energy industrial structure transformation in the local area, thus verifying H2 of this paper.

Panel B of Table 7 reports the reduction effect of digital finance on CO_2_ emissions by increasing green patents. As can be seen from the table, digital finance contributes to an increase in green patents granted at the 1% significance level when using the SAR model, while the result is positive at the 5% significance level when choosing the SDM. Thus, digital finance reduces CO_2_ emissions by promoting an increase in the number of green patents granted.

Panel C of Table 7 represents the reduction effect of digital finance on CO_2_ emissions through the promotion of green technological progress. As can be seen from the table, digital finance promotes green technological progress at the 5% significance level, but shows the siphon effect in the peripheries, suppressing the progress of green technology in the surrounding areas. Thus, digital finance reduces CO_2_ emissions in the local area by promoting green technological progress, but it does reflect the siphon effect, reducing green progress in surrounding areas and thus increasing carbon emissions in surrounding areas. Thus, it verifies H3 of this paper.

## 5. Further Discussion

Furthermore, in the multi-dimensional financial services of digital financial inclusion such as the breadth of coverage, depth of usage, and the level of digitization that enhances the development of finance, the roles of the different dimensions are not the same. In order to better investigate which dimension of digital finance contributes to carbon emission reduction better, this paper conducted the sub-hierarchical indicators test by adopting the SAR model. At the same time, although this paper has demonstrated that digital finance promotes carbon emission reduction above, whether the effect of digital finance shows a marginal diminishing effect in regions where digital finance itself is developed, or where economic development, green development, as well as green finance are developed, making its role weaker with the development of the above variables and hindering the further role of digital finance on carbon emission reduction is of great importance to explore. Thus, this paper also conducted sub-threshold tests through SAR model.

### 5.1. Sub-Hierarchical Indicators Test

Table 8 reports the regression results of the sub-hierarchical indicators test. From the second hierarchical index, the regression results of the use depth of digital finance (*USE*) on CO_2_ emissions are significantly negative at the level of 1%, and the regression results of digitization (*DIG*) on CO_2_ emissions are significantly negative at the level of 1%, while the regression results of the coverage breadth (*COV*) on CO_2_ emissions are not significant, indicating that the digitalization and usage depth of digital finance significantly inhibit CO_2_ emissions instead of coverage breadth. Among the third-hierarchical indicators, the regression of digital payment (*PAY*), insurance development (*INS*), and credit business (*CRE*) to CO_2_ emissions are significantly negative at the level of 1%, indicating that digital finance has increased the proportion of digital payments, created digital transaction scenarios, enhanced financial security, and made outstanding contributions to alleviating the financial constraints of regional high-tech and low-energy-consumption enterprises. 

By enhancing the convenience of low-carbon product transactions through digital transactions, guaranteeing digital transactions, the innovative operation of enterprises with digital financial insurance, as well as mitigating financial constraints, low-energy-consumption enterprises can better carry out energy industrial structure transformation and green technology upgrading, so as to reduce CO_2_ emissions.

### 5.2. Sub-Threshold Heterogeneity Test

In addition to the above baseline regression, endogenous test, robustness test, mediating mechanism test, and sub-hierarchical indicators test, this paper also carried out the sub-threshold heterogeneity test. Table 9 shows the regression results. Firstly, adopting the core explanatory variable itself as the threshold variable to distinguish the digital financial inclusion index, and the financial regulation (*FR*), i.e., the ratio of financial regulation expenditure to the regional financial added value, was also used to distinguish the threshold. The threshold value of the digital financial inclusion index was 237.53, while the threshold of financial regulation is 0.07. From columns (1) and (2) of Table 9, it can be seen that the higher the development of digital financial inclusion index, and the higher the ratio of financial regulation expenditure, the greater the reduction effect of digital finance on CO_2_ emissions in number, and the suppression effect is significant at the 1% level.

Secondly, this paper adopted the regional green development index (*GDI*) released by the National Bureau of Statistics of China as the threshold, and obtained the threshold value of 0.491, which shows that the effect of digital finance on CO_2_ emission reduction is significantly negative at 5% when it is less than the threshold value, and negative at 1% when it is greater than the threshold value, indicating that the higher the level of regional green development, the more significant the effect of digital finance on CO_2_ emission reduction.

Thirdly, regional heterogeneity was tested. The *North* variable was set to 1 when the location of the provincial capital was located north of the Kunlun Mountains–Chin ling Mountains–Huai River line and 0 otherwise. Column (4) of Table 9 reports the results of the test, showing that the effect of digital finance on CO_2_ reduction is larger numerically and significant at 1% in the south of China, while smaller numerically and not significant in the north. Since it is more developed in the south of China in terms of the economy overall, we can conclude that in economically developed regions, the effect of digital finance on carbon emissions is more significant.

Finally, this paper adopted the regional green finance index issued by the IIGF of the Central University of Finance and Economics as the threshold to distinguish the digital financial inclusion index and calculated so as to obtain two threshold values, which are 0.103 and 0.149, respectively. The regression result is reported in column (5) of Table 9, from which it can be seen that the effect of digital finance on CO_2_ emissions is not significant when the green finance index is lower than 0.103, and the effect of digital finance on CO_2_ emissions is significantly negative at the 5% significance level when the green finance index is between 0.103 and 0.149, while the effect of digital finance on CO_2_ emissions is significantly negative at the 1% significance level when the green finance index is higher than 0.149, and the reduced effect also increases numerically with higher development of green finance. Therefore, with the development of green finance, the effect of digital finance on carbon emission reduction is greater and more significant.

All in all, from the tests above, it can be seen that there is no marginal diminishing effect of digital finance on carbon emissions with the development of itself, economic level, green development, green finance, and increased expenditure in financial regulations. Therefore, promoting the development of digital finance could contribute to carbon emission reduction better. At the same time, digital finance can also help to reduce carbon emissions better in regions with better green development, more developed green finance or more developed economy as well as expending more in financial regulations.

## 6. Conclusions and Policy Recommendations

This paper used Chinese provincial panel data from 2011 to 2019, measured CO_2_ emissions of provinces in China by using the IPCC method as well as adopting The Peking University Digital Financial Inclusion Index of China, and explored the impact of digital finance on CO_2_ emissions through the SAR model and SDM. The following conclusions were drawn: 1. Digital finance significantly reduces CO_2_ emissions, and this result holds after using a 2SLS, dynamic spatial panel data model, spatial DID and spatial DDD exogenous shock tests, replacement model and matrix robustness tests, as well as replacement explained variable robustness tests. 2. Digital finance reduces CO_2_ emissions by promoting energy industrial structure transformation and spreads to surrounding areas through spillover effects. 3. Digital finance significantly contributes to the increase of green patents granted and thus reduces regional CO_2_ emissions. 4. Digital finance reduces local CO_2_ emissions by advancing green technological progress but reduces the green technological progress in surrounding areas and increases CO_2_ emissions due to the siphon effect. 5. Sub-hierarchical indicators tests show that the usage depth of digital finance, the degree of digitalization, the development of payment, insurance and credit businesses significantly suppress CO_2_ emissions; however, the effect of the breadth of coverage on CO_2_ emissions is not significant. 6. With the development of digital finance itself, the level of financial regulation, green development and the green finance index are higher, and the effect of digital finance on CO_2_ emission reduction is better. Digital finance significantly reduces CO_2_ emissions in the south of China, but not significantly in the north.

Based on the above analysis and conclusions, this paper maintains the following policy suggestions: 

First, vigorously promote the development of digital finance, advance the environmental protection undertakings and high-quality economic development through technological innovation and low-carbon emission reduction through the development of digital finance. Specifically, we can rely on big data, blockchain, and internet technology to promote financial support for green enterprises and projects and give full play to the ability to reach traditional financial inclusion that cannot be achieved by relying on digital platforms, so that green enterprises that have difficulty enjoying traditional financial services can enjoy the convenience of financial services through digital finance, thus promoting their development and upgrade, while fully encouraging digital platform lending institutions to alleviate the financing difficulties of green industry enterprises, improving the risk prevention of digital platform lending institutions, creating a good market environment, encouraging the innovation of financial services and products, and supporting the development of low-energy-consumption industries through the improvement of financial services. 

Second, strengthen the organic combination of deepening adjustment of industrial structure and adjustment of energy production and consumption structure, actively promote the transformation of energy consumption structure, realize the transformation from resource dependence to technology dependence, and realize the renewable use of energy and recycling of resources. Promote the ecological development of industries and industrial parks to reduce the intensity of energy and resource consumption. Coordinate and develop the green and low-carbon transformation of energy, and do a good job of green development, low-carbon development and circular development. 

Third, promote the transaction between carbon emissions of enterprises through digital platforms, provide carbon trading information for enterprises through the network unified trading platform based on big data and the Internet, alleviate the asymmetry of trading information, so as to strengthen the material return to low-carbon emission enterprises and improve the terminal gain of low-carbon action. 

Fourth, coordinate the improvement of energy efficiency and the development of non-fossil energy sources, such as putting the responsibility of increasing the proportion of non-fossil energy sources into the hands of local governments, and encouraging the transmission of non-fossil energy from regions with abundant non-fossil energy resources to regions with insufficient resources. This will allow for constructing channels for the eastward transmission of electricity from the west and the southward transmission of electricity from the north, taking advantage of the renewable energy resources in the northwest, and achieving a regional balance for sustainable development. 

This study has conducted a causal inference on the relationship between digital finance and carbon emission reduction by adopting panel data and excluding the endogenous problem, and also explored mediating mechanisms. However, firstly, in Section 2, this paper divided the enterprises into large-scale enterprises of energy-intensive industries and small-scale enterprises with low energy consumption. Digital finance promotes carbon emission reductions by easing financial constraints and supporting the development of SMEs, but for large-scale enterprises with low-energy-consumption, there are relatively few analyses. Secondly, the data in this study are at the provincial level of China, which is a small sample and geographically specific. Thus, future studies can consider using data from prefecture-level cities or countries around the world. Thirdly, in terms of peak carbon emissions, the indicator of it selected in this paper is the reduction of carbon emissions, which can be characterized by a more comprehensive hand. Finally, the non-linear correlation between digital finance and carbon emission reduction can also be explored.

## Figures and Tables

**Table 1:** Moran index tests.

Matrix	Provincial Adjacency		Provincial Capital Distance		Spatial Geographic Reverse	
*Variable*	*CO*	*DFI*	*CO*	*DFI*	*CO*	*DFI*
2011	0.152 ** (1.720)	0.473 *** (4.130)	0.1444 ** (1.917)	0.437 *** (4.008)	0.069 *** (3.288)	0.089 *** (3.818)
2012	0.135 * (1.560)	0.465 *** (4.111)	0.138 ** (1.853)	0.401 *** (4.619)	0.066 *** (3.180)	0.104 *** (4.328)
2013	0.133 * (1.536)	0.439 *** (3.926)	0.146 ** (1.935)	0.378 *** (4.404)	0.065 *** (3.128)	0.098 *** (4.162)
2014	0.125 * (1.465)	0.429 *** (3.824)	0.145 ** (1.926)	0.380 *** (4.432)	0.065 *** (3.140)	0.095 *** (4.060)
2015	0.103 * (1.254)	0.399 *** (3.596)	0.133 ** (1.785)	0.326 *** (3.856)	0.063 *** (3.041)	0.071 *** (3.320)
2016	0.133 * (1.528)	0.419 *** (3.773)	0.149 ** (1.925)	0.365 *** (4.283)	0.065 *** (3.120)	0.096 *** (4.117)
2017	0.112 * (1.341)	0.484 *** (4.320)	0.146 ** (1.928)	0.374 *** (1.386)	0.060 *** (2.960)	0.090 *** (3.927)
2018	0.1388 * (1.573)	0.538 *** (4.727)	0.149 ** (1.946)	0.396 *** (4.580)	0.057 ** (2.867)	0.096 *** (4.090)
2019	0.120 * (0.079)	0.536 *** (4.702)	0.134 ** (1.798)	0.407 *** (4.684)	0.055 ** (2.805)	0.099 ** (4.163)

Note: standardized Moran indices are in parentheses. ***, **, and * represent significance levels of 1%, 5%, and 10%, respectively.

**Table 2:** Descriptive statistics of main variables.

Variable	Observations	Mean	Std. Dev.	Minimum	Maximum
*CO*	270	5.535	0.722	3.488	6.798
*DFI*	270	203.358	91.568	18.33	410.281
*PGDP*	270	9.802	0.851	7.421	11.587
*POP*	270	8.204	0.735	6.342	9.352
*FEP*	270	4.782	0.620	3.055	6.617
*IX*	270	8.023	1.549	3.627	11.181
*IS*	270	2.366	0.129	2.166	2.832
*UG*	270	39.367	3.574	27.9	49.1
*ES*	270	0.538	0.210	0.196	1.161
*GP*	270	0.374	0.499	0.002	3.08
*TP*	270	74.922	0.113	74.689	75.127

**Table 3:** Regression results of digital financial inclusion index on CO_2_ emissions.

	(1)	(2)	(3)	(4)	(5)	(6)
Model	SAR	SDM Main	SDM Spatial	SAR	SDM Main	SDM Spatial
*DFI*	−0.0024 ***	−0.0031 **	0.0077	−0.0029 ***	−0.0026 **	0.0059
	(0.0008)	(0.0013)	(0.0077)	(0.0010)	(0.0013)	(0.0109)
*PGDP*				−0.0397	−0.0329	0.3667
				(0.0935)	(0.0909)	(0.4662)
*POP*				2.3623 *	2.4223 *	−5.8118
				(1.3162)	(1.4674)	(5.1534)
*FEP*				0.0043	0.0080	−0.0179
				(0.0089)	(0.0081)	(0.0615)
*IX*				−0.0487	−0.0478	0.0092
				(0.0372)	(0.0470)	(0.2779)
*IS*				−0.2483	−0.2173	2.9673
				(0.3227)	(0.3173)	(2.0864)
*UG*				−0.0091	−0.0065	0.0675
				(0.0070)	(0.0068)	(0.0447)
*WCO*	−0.5589 *	−0.5346 ***		−0.4258 *	−0.2676 *	
	(0.3262)	(0.1898)		(0.2493)	(0.1513)	
Province Effect	Fixed	Fixed		Fixed	Fixed	
Time Effect	Fixed	Fixed		Fixed	Fixed	
R-squared	0.0603	0.0768		0.5770	0.5295	

Note: Robust standard errors clustering to province-level are in parentheses. ***, **, and * represent significance levels of 1%, 5%, and 10%, respectively.

**Table 4:** Regression results of endogenous tests.

Panel A	(1)	(2)	(3)	(4)	(5)	(6)
Model	SAR	SDM Main	SDM Spatial	SAR	SDM Main	SDM Spatial
*DIS*	0.0580 ***	0.0433 *	0.1818 *			
	(0.0216)	(0.0233)	(0.1091)			
*TS*				0.1239 ***	0.0730 ***	0.5673 ***
				(0.0437)	(0.0279)	(0.1721)
*WCO*	0.4063 **	0.3746 *** (0.1280)		0.4426 ***	0.3483 *** (0.1197)	
	(0.1833)			(0.1640)		
R-squared	0.2909	0.7755		0.9071	0.8326	
**Panel B**	**(1)**	**(2)**	**(3)**	**(4)**	**(5)**	**(6)**
Instrumental variable		*DIS*			*TS*	
model	SAR	SDM Main	SDM Spatial	SAR	SDM Main	SDM Spatial
*DFI*	−0.0019 ***	−0.0018 **	−0.0038	−0.0099 **	−0.0099 **	0.0309
	(0.0005)	(0.0007)	(0.0041)	(0.0040)	(0.0046)	(0.0391)
*WCO*	−0.5053 (0.3161)	−0.3075 (0.3007)		−0.4422 * (0.2594)	−0.2495 (0.1820)	
R-squared	0.5784	0.4990		0.5809	0.5108	
**Panel C**	**(1)**	**(2)**	**(3)**	**(4)**	**(5)**	**(6)**
Model	SAR	SDM Main	SDM Spatial	SAR	SDM Main	SDM Spatial
*CO*				0.6125 ***	0.6491 ***	−1.9753 ***
				(0.0819)	(0.0820)	(0.7578)
*DFI*	−0.0050 **	−0.0048 **	0.0033	−0.0032 ***	−0.0030 **	−0.0051
	(0.0023)	(0.0024)	(0.0117)	(0.0012)	(0.0014)	(0.0082)
*WCO*	0.6891 ***	0.4313 *** (0.1592)		0.7341 ***	0.5573 *** (0.1343)	
	(0.1008)			(0.0834)		
R-squared	0.5089	0.3431		0.6901	0.3844	
Covariables	Yes	Yes	Yes	Yes	Yes	Yes
Province effect	Fixed	Fixed		Fixed	Fixed	
Time effect	Fixed	Fixed		Fixed	Fixed	

Note: ***, **, and * represent significance levels of 1%, 5%, and 10%, respectively.

**Table 5:** Regression results of exogenous shock test.

	(1)	(2)	(3)	(4)	(5)	(6)
Model	SAR-DID	SDM Main	SDM Spatial	SAR-DDD	SDM Main	SDM Spatial
*DDD*				−0.0707 *	−0.0902 **	0.0616
				(0.0403)	(0.0422)	(0.1085)
*DID*	−0.0889 **	−0.0781 **	−0.1898	−0.0304	−0.0177	−0.1092
	(0.0372)	(0.0360)	(0.3404)	(0.0293)	(0.0297)	(0.0833)
*POST* × *SECT*				0.0388	0.0571 *	−0.0143
				(0.0296)	(0.0304)	(0.0772)
*TREAT* × *SECT*				−0.3116	−0.0425	−1.1986 **
				(0.2941)	(0.2271)	(0.5845)
*TREAT*				0.2163	0.1073	0.2824
				(0.2073)	(0.1613)	(0.4629)
*POST*				0.0197	0.0124	0.0182
				(0.0273)	(0.0546)	(0.0000)
*SECT*				0.6393 ***	0.5842 ***	1.7031 ***
				(0.2030)	(0.1454)	(0.3819)
Covariables	Yes	Yes	Yes	Yes	Yes	Yes
*WCO*	−0.4077 *	−0.2938 * (0.1750)		0.1954	0.2050 * (0.1113)	
	(0.2298)			(0.1842)		
Constant				−1.3923	3.4251 * (2.0717)	
				(1.4010)		
Province Effect	Fixed	Fixed		Random	Random	
Time Effect	Fixed	Fixed		Random	Random	
R-squared	0.5826	0.5302		0.7232	0.8673	

Note: ***, **, and * represent significance levels of 1%, 5%, and 10%, respectively.

**Table 6:** Robustness tests.

Panel A	(1)	(2)	(3)	(4)	(5)	(6)
Model	Pooled OLS	FE	DIF-GMM	SYS-GMM	SEM	SAC
*CO*			0.1959	0.1959		
			(0.3806)	(0.2575)		
*DFI*	−0.0022 **	−0.0029 ***	−0.0047 **	−0.0047 **	−0.0029 ***	−0.0029 ***
	(0.0008)	(0.0010)	(0.0021)	(0.0020)	(0.0010)	(0.0009)
*WCO*					−0.4108 *	−0.4108
					(0.2425)	(0.3568)
*lambda*					−0.0468	−0.0468
					(0.3379)	(0.2753)
Constant	−1.6786	−12.1009	−25.6552 **	−26.1889 ***		
	(3.0739)	(10.4182)	(12.9499)	(8.6343)		
R-squared	0.7041	0.5823	None	None	0.5767	0.5767
**Panel B**	**(1)**	**(2)**	**(3)**	**(4)**	**(5)**	**(6)**
Weighted Matrix	Provincial Adjacency Matrix			Provincial Capital Distance Matrix		
Model	SAR	SDM Main	SDM Spatial	SAR	SDM Main	SDM Spatial
*DFI*	−0.0030 ***	−0.0034 ***	0.0032	−0.0030 ***	−0.0023 **	0.0024
	(0.0009)	(0.0013)	(0.0021)	(0.0009)	(0.0011)	(0.0022)
*WCO*	0.3373 ***	0.3539 ***		0.1263	0.1579	
	(0.1132)	(0.0963)		(0.0992)	(0.1135)	
R-squared	0.4647	0.5424		0.5485	0.5830	
**Panel C**	**(1)**	**(2)**	**(3)**	**(4)**	**(5)**	**(6)**
Explained Variable	Raw value of CO_2_ emissions			Data from CEADs		
Model	SAR	SDM Main	SDM Spatial	SAR	SDM Main	SDM Spatial
*DFI*	−0.0009 **	−0.0013 **	0.0037	−0.0036 ***	−0.0034 ***	0.0020
	(0.0004)	(0.0006)	(0.0034)	(0.0011)	(0.0010)	(0.0106)
*WCO*	−0.7085 **	−0.6534 **		−0.1867	−0.1962	
	(0.3030)	(0.2993)		(0.2184)	(0.1601)	
R-squared	0.4078	0.4135		0.5865	0.6330	
Covariables	Yes	Yes	Yes	Yes	Yes	Yes
Province Effect	Fixed	Fixed		Fixed	Fixed	
Time Effect	Fixed	Fixed		Fixed	Fixed	

Note: ***, **, and * represent significance levels of 1%, 5%, and 10%, respectively.

**Table 7:** Mediating mechanisms tests.

Panel A	(1)	(2)	(3)		(4)	(5)	(6)
Explained Variable	*ES*				*CO*		
Model	SAR	SDM Main	SDM Spatial		SAR	SDM Main	SDM Spatial
*DFI*	−0.0025 ***	−0.0015 *	−0.0132 ***				
	(0.0009)	(0.0009)	(0.0049)				
*ES*					0.3603 ***	0.3437 ***	0.3233
					(0.0745)	(0.0738)	(0.5534)
*WES*	−0.7045 ***	−0.7860 **					
	(0.2383)	(0.3141)					
*WCO*					−0.5166	−0.3882	
					(0.3150)	(0.3122)	
R-squared	0.4864	0.4507			0.6085	0.5010	
**Panel B**	**(1)**	**(2)**		**(3)**	**(4)**	**(5)**	**(6)**
Explained Variable	*GP*				*CO*		
Model	SAR	SDM Main		SDM Spatial	SAR	SDM Main	SDM Spatial
*DFI*	0.0138 ***	0.0102 **		0.0225			
	(0.0040)	(0.0041)		(0.0280)			
*GP*					−0.0817 ***	−0.0746 ***	0.0861
					(0.0222)	(0.0252)	(0.1564)
*WGP*	0.2640	0.0626 (0.2498)					
	(0.2035)						
*WCO*					−0.4386	−0.2903 (0.3026)	
					(0.3097)		
R-squared	0.3027	0.2460			0.5828	0.5302	
**Panel C**	**(1)**	**(2)**		**(3)**	**(4)**	**(5)**	**(6)**
Explained Variable		*TP*				*CO*	
Model	SAR	SDM Main		SDM Spatial	SAR	SDM Main	SDM Spatial
*DFI*	0.0005 **	0.0004 **		−0.0038 ***			
	(0.0002)	(0.0002)		(0.0011)			
*TP*					−4.7557 ***	−4.7411 ***	−0.6513
					(0.7215)	(0.7345)	(5.5429)
*WTP*	−0.6107	−0.7381 *					
	(0.4335)	(0.4106)					
*WCO*					−0.3464	−0.2387	
					(0.2969)	(0.2905)	
R-squared	0.7825	0.9063			0.5742	0.4343	
Covariables	Yes	Yes		Yes	Yes	Yes	Yes
Province Effect	Fixed	Fixed			Fixed	Fixed	
Time Effect	Fixed	Fixed			Fixed	Fixed	

Note: ***, **, and * represent significance levels of 1%, 5%, and 10%, respectively.

**Table 8:** Sub-hierarchical indicators test.

	(1)	(2)	(3)	(4)	(5)	(6)
*USE*	−0.0019 ***					
	(0.0006)					
*COV*		0.0033				
		(0.0028)				
*DIG*			−0.0009 ***			
			(0.0003)			
*PAY*				−0.0013 ***		
				(0.0005)		
*INS*					−0.0006 ***	
					(0.0002)	
*CRE*						−0.0147 ***
						(0.0033)
Covariables	Yes	Yes	Yes	Yes	Yes	Covariables
*WCO*	−0.4365 *	−0.5233 **	−0.4888 **	−0.4494	−0.4626 *	0.2148
	(0.2506)	(0.2391)	(0.2357)	(0.3116)	(0.2633)	(0.2577)
Province Effect	Fixed	Fixed	Fixed	Fixed	Fixed	Fixed
Time Effect	Fixed	Fixed	Fixed	Fixed	Fixed	Fixed
R-squared	0.5842	0.5779	0.5833	0.5862	0.5830	0.3281

Note: ***, **, and * represent significance levels of 1%, 5%, and 10%, respectively.

**Table 9:** Sub-threshold heterogeneity test of the effect of digital finance on CO_2_ emissions.

	(1)	(2)	(3)	(4)	(5)
Threshold Variable	*DFI*	*FR*	*GDI*	*North*	*GFI*
Threshold Value	237.53	0.007	0.491	0.5	0.103; 0.149
*DFI* low	−0.0030 ***	−0.0026 ***	−0.0025 **	−0.0049 ***	−0.0011
	(0.0009)	(0.0010)	(0.0010)	(0.0016)	(0.0014)
*DFI* middle					−0.0032 **
					(0.0014)
*DFI* high	−0.0033 ***	−0.0030 ***	−0.0027 ***	−0.0005	−0.0037 ***
	(0.0010)	(0.0009)	(0.0010)	(0.0015)	(0.0013)
Covariables	Yes	Yes	Yes	Yes	Yes
*WCO*	11.9629 **	12.9309 **	12.1240 **	12.8181 **	−6.5432 ***
	(5.5214)	(5.4806)	(5.5584)	(5.5991)	(0.6023)
Province Effect	Fixed	Fixed	Fixed	Fixed	Fixed
Time Effect	Fixed	Fixed	Fixed	Fixed	Fixed
R-squared	0.6144	0.6028	0.6060	0.6063	0.4018

Note: Standard errors are in parentheses. *** and ** represent significance levels of 1% and 5%, respectively.

## Data Availability

Not applicable.

## References

[1] Cong J., Pang T., Peng H. (2020). Optimal Strategies for Capital Constrained Low-Carbon Supply Chains under Yield Uncertainty. J. Clean. Prod..

[2] Le Quéré C., Korsbakken J.I., Wilson C., Tosun J., Andrew R., Andres R.J. (2019). Drivers of declining CO_2_ emissions in 18 developed economies. Nat. Clim. Chang..

[3] Eyring V., Cox P.M., Flato G.M., Gleckler P.J., Abramowitz G., Caldwell P. (2019). Taking climate model evaluation to the next level. Nat. Clim. Chang..

[4] Intergovernmental Panel on Climate Change Special Report on Global Warming of 1.5 °C. https://www.ipcc.ch/sr15/.

[5] Kemp L. (2017). Better out than in. Nat. Clim. Chang..

[6] United Nations Framework Convention on Climate Change (UNFCCC) (2015). Enhanced Actions on Climate Change: China’s Intended Nationally Determined Contributions. https://unfccc.int.

[7] Yu M.L., Tsai F.S., Jin H., Zhang H.J. (2022). Digital finance and renewable energy consumption: Evidence from China. Financ. Innov..

[8] Aziz A., Naima U. (2021). Rethinking digital financial inclusion: Evidence from Bangladesh. Technol. Soc..

[9] Shahbaz M., Li K., Dong X., Dong K. (2022). How financial inclusion affects the collaborative reduction of pollutant and carbon emissions: The case of China. Energy Econ..

[10] Wen H., Yue J., Li J., Xiu X., Zhong S. (2022). Can digital finance reduce industrial pollution? New evidence from 260 cities in China. PLoS ONE.

[11] Wang H., Guo J. (2022). Impacts of digital inclusive finance on CO_2_ emissions from a spatial perspective: Evidence from 272 cities in China. J. Clean. Prod..

[12] Tian X., Zhang Y., Qu G. (2022). The Impact of Digital Economy on the Efficiency of Green Financial Investment in China’s Provinces. Int. J. Environ. Res. Public Health.

[13] Gao Q., Chen C., Sun G., Li J. (2022). The Impact of Digital Inclusive Finance on Agricultural Green Total Factor Productivity: Evidence from China. Front. Ecol. Evol..

[14] Xie C., Liu C. (2022). The Nexus between Digital Finance and High-Quality Development of SMEs: Evidence from China. Sustainability.

[15] Elheddad M., Benjasak C., Deljavan R., Alharthi M., Almabrok J.M. (2021). The effect of the Fourth Industrial Revolution on the environment: The relationship between electronic finance and pollution in OECD countries. Technol. Forecast. Soc. Chang..

[16] Guo F., Wang J.Y., Wang F., Kong T., Zhang X., Cheng Z.Y. (2020). Measuring the Development of Digital financial inclusion in China: Index Compilation and Spatial Characteristics. China Econ. Q..

[17] Asongu A.S., Biekpe N., Cassimon D. (2021). On the diffusion of mobile phone innovations for financial inclusion. Technol. Soc..

[18] Liu G., Huang Y.Y., Huang Z.H. (2021). Determinants and Mechanisms of Digital Financial Inclusion Development: Based on Urban-Rural Differences. Agronomy.

[19] Tay L., Tai H., Tan G. (2022). Digital financial inclusion: A gateway to sustainable development. Heliyon.

[20] Wang X., Wang X., Ren X.H., Wen F.H. (2022). Can digital financial inclusion affect CO_2_ emissions of China at the prefecture level? Evidence from a spatial econometric approach. Energy Econ..

[21] Dai D., Fan Y., Wang G., Xie J. (2022). Digital Economy, R & D Investment, and Regional Green Innovation—Analysis Based on Provincial Panel Data in China. Sustainability.

[22] Kong T., Sun L., Sun G., Song Y. (2022). Effects of Digital Finance on Green Innovation considering Information Asymmetry: An Empirical Study Based on Chinese Listed Firms. Emerg. Mark. Financ. Trade.

[23] Duarte J., Siegel S., Young L. (2012). Trust and Credit: The Role of Appearance in Peer-to-Peer Lending. Rev. Financ. Stud..

[24] Luo S. (2022). Digital Finance Development and the Digital Transformation of Enterprises: Based on the Perspective of Financial constraint and Innovation Drive. J. Math..

[25] Moenninghoff S.C., Wieandt A. (2013). The Future of Peer-to-Peer Finance.

[26] Feng S.L., Zhang R., Li G.X. (2022). Environmental decentralization, digital finance and green technology innovation. Struct. Chang. Econ. Dyn..

[27] Tobelmann D., Wendler T. (2020). The impact of environmental innovation on carbon dioxide emissions. J. Clean. Prod..

[28] Svirydzenka K. (2016). Introducing a New Broad-Based Index of Financial Development.

[29] Ma L.M., Zhang X. (2014). Spatial Effects of Haze Pollution and the Impact of Economic and Energy Structure in China. China Ind. Econ..

[30] Battese G.E., O’Donnell C.J. (2004). A Metafrontier Production Function for Estimation of Technical Efficiencies and Technology Gaps for Firms Operating under Different Technologies. J. Product. Anal..

[31] Zhang J., Wu G.Y., Zhang J.P. (2004). Estimation of China’s Provincial Capital Stock: 1952~2000. Econ. Res. J..

[32] Cao S., Nie L., Sun H., Sun W., Taghizadeh-Hesary F. (2021). Digital finance, green technological innovation and energy-environmental performance: Evidence from China’s regional economies. J. Clean. Prod..

[33] Zhang W., Liu X., Wang D., Zhou J. (2022). Digital economy and carbon emission performance: Evidence at China’s city level. Energy Policy.

[34] Bullock J.G., Green D.P., Ha S.E. (2010). Yes, But What’s the Mechanism? (Don’t Expect an Easy Answer). J. Personal. Soc. Psychol..

[35] Zhang X., Yang T., Wang C., Wan G.H. (2020). Digital Financial Development and Household Consumption Growth: Theory and China’s Practice. Manag. World.

[36] Liu T., Xu Z.Y., Zhang K.L. (2022). The Impact of Digital Finance on the Synergy of Economic Development and Ecological Environment. Mod. Financ. Econ..

[37] Song M., Zhou P., Si H.T. (2021). Financial Technology and Enterprise Total Factor Productivity—Perspective of “Enabling” and Credit Rationing. China Ind. Econ..

[38] Qi S.Z., Zhou C.B., Li K., Tang S.Y. (2021). The impact of a carbon trading pilot policy on the low-carbon international competitiveness of industry in China: An empirical analysis based on a DDD model. J. Clean. Prod..

